# The cuproptosis-related signature associated with the tumor environment and prognosis of patients with glioma

**DOI:** 10.3389/fimmu.2022.998236

**Published:** 2022-08-30

**Authors:** Weichen Wang, Zhichao Lu, Maoyu Wang, Zongheng Liu, Bing Wu, Chengkai Yang, He Huan, Peipei Gong

**Affiliations:** Department of Neurosurgery, Affiliated Hospital of Nantong University, Medical School of Nantong University, Nantong, China

**Keywords:** glioma, cuproptosis, signature, clusters, bioinformatics

## Abstract

**Background:**

Copper ions are essential for cellular physiology. Cuproptosis is a novel method of copper-dependent cell death, and the cuproptosis-based signature for glioma remains less studied.

**Methods:**

Several glioma datasets with clinicopathological information were collected from TCGA, GEO and CGGA. Robust Multichip Average (RMA) algorithm was used for background correction and normalization, cuproptosis-related genes (CRGs) were then collected. The TCGA-glioma cohort was clustered using ConsensusClusterPlus. Univariate Cox regression analysis and the Random Survival Forest model were performed on the differentially expressed genes to identify prognostic genes. The cuproptosis-signature was constructed by calculating CuproptosisScore using Multivariate Cox regression analysis. Differences in terms of genomic mutation, tumor microenvironment, and enrichment pathways were evaluated between high- or low-CuproptosisScore. Furthermore, drug response prediction was carried out utilizing pRRophetic.

**Results:**

Two subclusters based on CRGs were identified. Patients in cluster2 had better clinical outcomes. The cuproptosis-signature was constructed based on CuproptosisScore. Patients with higher CuproptosisScore had higher WHO grades and worse prognosis, while patients with lower grades were more likely to develop IDH mutations or MGMT methylation. Univariate and Multivariate Cox regression analysis demonstrated CuproptosisScore was an independent prognostic factor. The accuracy of the signature in prognostic prediction was further confirmed in 11 external validation datasets. In groups with high-CuproptosisScore, PIK3CA, MUC16, NF1, TTN, TP53, PTEN, and EGFR showed high mutation frequency. IDH1, TP53, ATRX, CIC, and FUBP1 demonstrated high mutation frequency in low-CuproptosisScore group. The level of immune infiltration increased as CuproptosisScore increased. SubMap analysis revealed patients with high-CuproptosisScore may respond to anti-PD-1 therapy. The IC50 values of Bexarotene, Bicalutamide, Bortezomib, and Cytarabine were lower in the high-CuproptosisScore group than those in the low-CuproptosisScore group. Finally, the importance of IGFBP2 in TCGA-glioma cohort was confirmed.

**Conclusion:**

The current study revealed the novel cuproptosis-based signature might help predict the prognosis, biological features, and appropriate treatment for patients with glioma.

## Introduction

Glioma is the most common primary brain tumor, accounting for about 40% of all brain tumors (1), among which glioblastoma (GBM) is the most malignant brain tumor. According to the classification of World Health Organization (WHO), gliomas are classified into four different histopathological grades: Grade I, II, III and IV, of which WHO II and III are considered low-grade gliomas (LGG). Glioblastoma (GBM, WHO IV) characterized by new angiogenesis is the most aggressive molecular subtype of glioma (2, 3). The median survival of LGG can be achieved from five to ten years through the administration of surgery, radiotherapy, and chemotherapy combination treatments, whereas the median survival of GBM is normally around one or two years (4, 5). The prognosis of glioma patients is divergent, which may be related to different tumor grades, mutation of isocitrate dehydrogenase (IDH) (6), amplification of epidermal growth factor receptor (EGFR) (7) and other factors. The current glioma prognostic evaluation model is mainly based on clinical factors, which has limited predictive ability (8–11). Therefore, a better prognostic evaluation system is needed.

Gene-regulated cell death known as “programmed cell death” is crucial for tissue homeostasis and growth, it also takes part in several pathological processes (12). At present, various types of cell death, such as necroptosis, ferroptosis, and pyroptosis, have been found to belong to necrotic programmed cell death (12). Researchers have found that cell death is closely related to tumorigenesis and prognosis. In the process of tumor development, cell death often occurs in the intratumoral area due to metabolic stress, such as hypoxia or glucose deprivation (13). Consequently, triggering programmed cell death could be a potential strategy for novel tumor therapy. Currently, knowledge of programmed cell death in cancer is continuously updated as more types of programmed cell death are discovered and recognized. Biology has long recognized copper as a vital component in all living things, from bacteria and fungi to mammals and plants, where it is a must for survival (14, 15). In humans, it binds to enzymes that assist in blood clotting, hormone maturation, and cell processing of energy, however, excessive copper can cause cell death (14). Cuproptosis as a new type of cell death is modulated and regulated by copper in cells. Copper ion directly binds to the lipoacylated components in the tricarboxylic acid cycle, leading to abnormal aggregation of lipoacylated proteins and loss of iron-sulfur cluster proteins, which leads to protein toxic stress and ultimately leads to cell death (15). Cuproptosis in glioma, however, has not yet been studied in depth.

The establishment of a glioma prognosis prediction model based on transcriptome data combined with clinical data can improve the prediction ability to a certain extent, which has highly significant clinical significance. In this study, glioma gene expression data and clinical data were collected from open databases, combined with reported cuproptosis-related genes (CRGs), was used to establish and verify cuproptosis-related clusters and signature. Subsequently, the prognosis, immune status, and treatment response of patients was also explored based on the cuproptosis-related clusters and signature.

## Materials and methods

### Collection and preprocessing of data for glioma

Transcripts and clinical data of glioma samples, including survival status, IDH status, grade, gender, and age, were collected from TCGA database based on UCSC Xena platform (16, 17). A total of 656 glioma patients with corresponding data were enrolled. Meanwhile, the gene expression profile of the control (non-tumoral samples) were also obtained from Genome Tissue Expression (GTEx) project (https://www.gtexportal.org) (18, 19). In addition, 11 glioma-cohorts (CGGA311, CGGA668, GSE108474, GSE13041, GSE16011, GSE43289, GSE43378, GSE4412, GSE4412, GSE68838, and GSE83300) were collected from Gene Expression Omnibus (GEO, https://www.ncbi.nlm.nih.gov/geo/) or Chinese Glioma Genome Atlas (CGGA, http://www.cgga.org.cn/). Robust Multichip Average (RMA) algorithm was used for background correction and normalization (20). Data in the form of fragments per kilobase million (FPKM) was transformed into transcripts per kilobase million (TPM). The list of cuproptosis-related genes (CRGs) refers to the previous literature (14). In the end, ten CRGs were included in our study: FDX1, LIAS, LIPT1, DLD, DLAT, PDHA1, POHB, MTF1, GLS, and CDKN2A.

### Establishment of cuproptosis-clusters and cuproptosis-signature

Based on the collected ten CRGs, the TCGA-glioma cohort was clustered using ConsensusClusterPlus package (21). Next, principal component analysis (PCA) (22) was further carried out to assess patterns associated with cuproptosis. Limma package was used to identify the differentially expressed genes (DEGs) in cuproptosis-clusters (logFC>1, P<0.05) (23). Subsequently, Univariate Cox regression analysis was performed to identify prognostic DEGs preliminarily (24). Subsequently, more valuable prognostic genes were screened out based on Random Survival Forest model (variable importance>0.25) (25, 26). To construct a cuproptosis-signature, Multivariate Cox regression analysis was used to estimate and weight the regression coefficients of the prognostic genes, and the CuproptosisScore for each glioma sample was calculated. According to the best optimal cutoff, the patients were divided into high- or low-CuproptosisScore subgroups. The association between overall survival (OS) and CuproptosisScore was analyzed using Kaplan-Meier curves. ROC curves were further utilized to validate the efficiency and accuracy of CuproptosisScore in predicting outcomes at one-, two-, and three-year. In addition, Univariate or Multivariate Cox regression analyses of CuproptosisScore and several clinical factors were performed to verify the independence of CuproptosisScore in predicting prognosis.

### Genomic mutation analysis for cuproptosis-signature

The data of somatic mutations (16, 27) or copy number variation (CNV) (16) was acquired from TCGA. Genomic Identification of Significant Targets in Cancer (GISTIC) (28) algorithm was used to assess genomic characterization and CNV landscape.

### Analysis of immune infiltration

Immune cell abundance (immune score), stromal cell infiltrating level (stromal score), and tumor purity (ESTIMATE score) were estimated *via* ESTIMATE (The Estimation of Stromal and Immune cells in Malignant Tumor tissues using Expression) algorithm (29). Using Tumor Immune Estimation Resource 2.0 (TIMER 2.0, http://timer.cistrome.org/) (30), a comprehensive analysis of immune infiltration in the tumor microenvironment of glioma was carried out. MCPcounter algorithm was used to estimate the relative proportions of ten immune cells in glioma (31). The infiltration of 28 immune cells was indicated by enrichment scores, which were calculated by single sample gene set enrichment analysis (ssGSEA) using Gene Set Variation Analysis (GSVA) R package (32, 33). Immunomodulators associated with seven different immune processes (Antigen presentation, Cell adhesion, Co-inhibitor, Co-stimulator, Ligand, Receptor and Other) were obtained from the previous literature (34). The response of glioma to anti-PD1 and anti-CTLA4 therapy was evaluated by Submap algorithm (35–37).

### Enrichment pathway analysis

All gene sets from Gene Ontology (GO) and Kyoto Encyclopedia of Genes and Genomes (KEGG) were downloaded from the MSigDB database (38). Gene Set Enrichment Analysis (GSEA) (39) and Gene Set Variation Analysis (GSVA) (33) were carried out according to clusterProfiler and GSVA packages, respectively.

### Drug response prediction

Pharmacogenomic data from Genomics of Drug Sensitivity in Cancer (GDSC) (40) database was used to predict drug sensitivity in the enrolled glioma cases. The half maximal inhibitory concentration (IC50) value was calculated by pRRophetic package to reflect the drug response (41).

### Immunohistochemistry (IHC) staining

The tissue sections through deparaffinization and dehydration were incubated with polyclonal rabbit anti-human IGFBP2 antibodies (1:50, Proteintech, 11065-3-AP) overnight at 4°C after epitope retrieval, H2O2 treatment, and non-specific antigens blocking. Next, sections were incubated with secondary antibodies (1:1000, Proteintech, SA00001-2) for two hours at room temperature, and then the signal was detected by an enhanced DAB staining kit (Proteintech, China).

### Western blot

Tumor tissues as well as normal tissues were lysed in RIPA buffer (Solarbio, Beijing, China), protease and phosphatase inhibitors were added, and then denatured at 100°C for 15 min. The protein samples were then separated by 10% SDS-PAGE and transferred to polyvinylidene fluoride (PVDF) membranes. Next, PVDF membranes were blocked with 5% skim milk powder solution for 1 hour, and incubated with primary antibodies, including anti-IGFBP2 antibody (1:1000, Proteintech, 11065-3-AP), anti-GAPDH antibody (1:5000, Abcam, ab9485) overnight, followed by secondary antibodies (1:2000, Proteintech, SA00001-2) for 2 hours at room temperature, observed with the ECL kit chemiluminescence reagent (Billerica Millipore, MA, USA). Protein band signals were detected by the Chemidoc detection system (Bio-Rad, Hercules, CA, USA) and quantified by ImageJ software (National Institutes of Health, USA).

### Statistical analysis

The Wilcoxon test was used to compare non-normally distributed data. The T-test was used to compare normally distributed variables between two groups. The R package survminer was used to estimate OS between two groups using Kaplan-Meier survival plots. Cox regression of survival analysis was also performed by survival. Time-dependent receiver operating characteristics (ROC) curves were plotted using R package timeROC. All heatmaps were performed through pheatmap package. The data were visualized by ggplot2 (V4.1.2). P<0.05 was considered statistically significant.

## Results

### Characteristics of cuproptosis-clusters for TCGA-glioma

**Figure 1:**
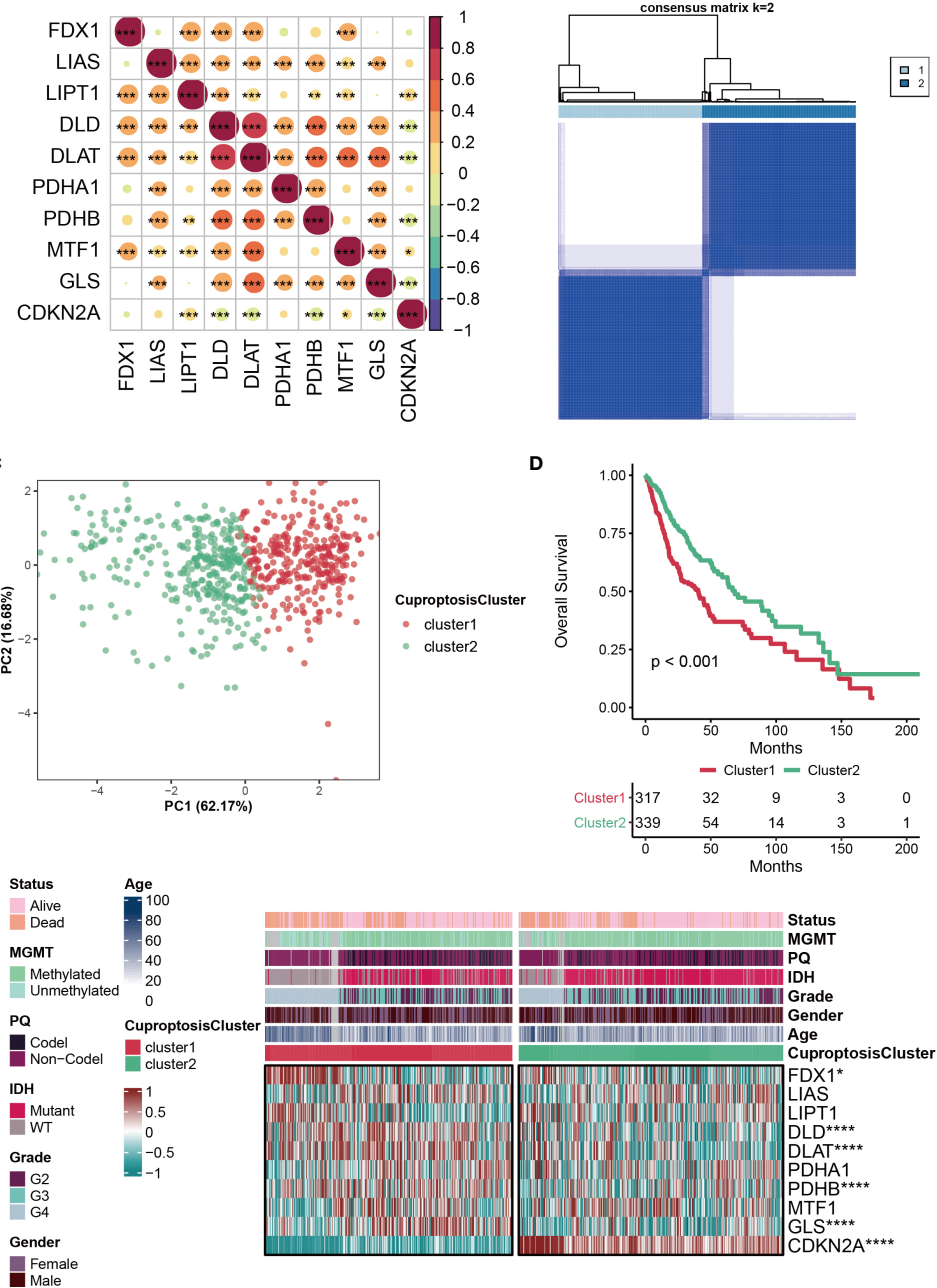
Characteristics of CuproptosisCluster in TCGA-glioma cohort. **(A)** The correlations among the ten cuproptosis-related genes (CRGs). The color represents the correlation coefficient. **(B)** Cluster diagram for subtype analysis of glioma samples. The intragroup correlations were the highest and the inter-group correlations were low when k=2. **(C)** PCA analysis for the two subclusters. **(D)** Kaplan-Meier survival curve showing survival probability of cluster1 and cluster2. **(E)** Heatmap showing the expression levels of the ten cuproptosis-related genes (CRGs) in different clinical features and clusters. *P < 0.05; **P < 0.01; ***P < 0.001; ****P < 0.0001.

The clinical information of patients from TCGA was listed in the **Supplemental Table S1**. The correlations among the ten CRGs were mostly positive, and the most strongly associated variables were DLD and DLAT **(** **Figure 1A** **)**. Based on ConsensusClusterPlus, the optimal number of clusters was determined, k=2 **(** **Figure 1B** **)**. Furthermore, PCA analysis was further used to validate that patients in the two subclusters clustered separately, which confirmed the reliability of the clustering results **(** **Figure 1C** **)**. Patients with glioma in cluster2 had significantly better clinical outcomes than those in cluster1 **(** **Figure 1D** **)**. Compared with the other subcluster, the expressions of FDX1, DLD, DLAT, PDHB, GLS were relatively high in cluster1, while the expression of CDKN2A was relatively high in cluster2, which indicates that these CRGs may be genetic markers for identifying different clusters **(** **Figure 1E** **)**. Interestingly, we discovered that cluster2 had a greater percentage of IDH mutation status, which may be one of the factors contributing to a better prognosis of this subcluster **(** **Figure 1E** **)**.

### Establishment of cuproptosis-signature

**Figure 2:**
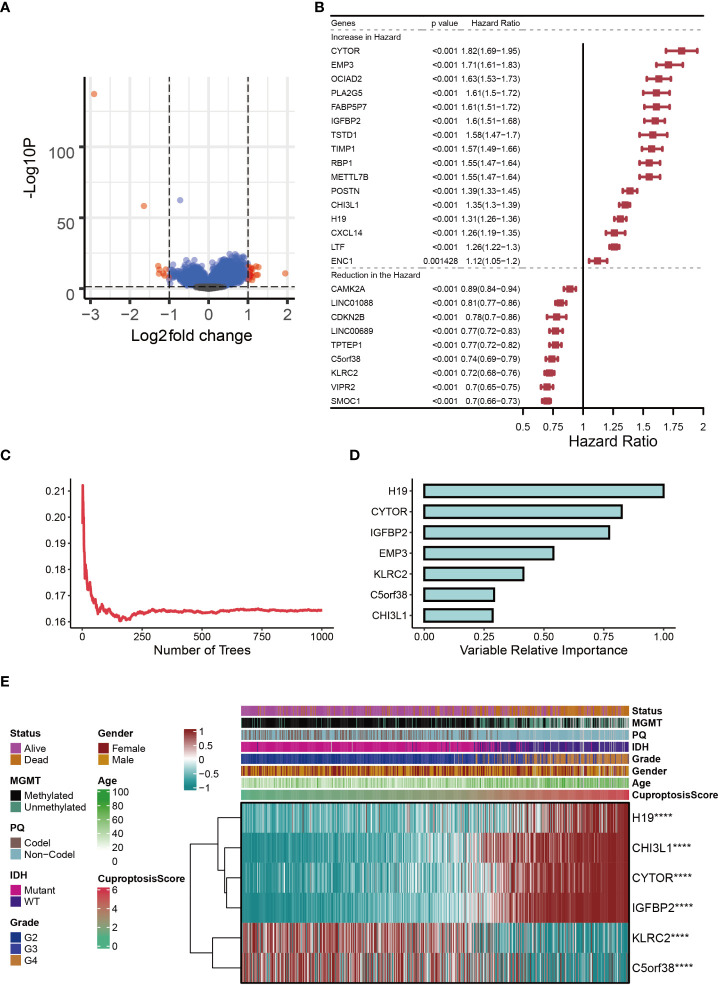
Establishment of cuproptosis-signature. **(A)** The volcano map reflects the differentially expressed genes identified (logFC > 1, P < 0.05). **(B)** The forest figure for Univariate Cox regression analysis of the differentially expressed genes. **(C)** The distribution of error rates in Random Survival Forest model. **(D)** The variable relative importance of the seven genes. **(E)** Heat map showing the relationship between six genes in the cuproptosis-signature and CuproptosisScore distribution and its clinical characteristics. ****P < 0.0001.

In the two subclusters, a total of 27 differentially expressed genes (DEGs) were identified (logFC>1, P<0.05), and the volcano map accurately reflected the gene expression differences between the two subclusters **(** **Figure 2A** **)**. After Univariate Cox regression analysis, 16 potential pro-oncogenes (HR>1; CYTOR, EMP3, OCIAD2, PLA2G5, FABP5P7, IGFBP2, TSTD1, TIMP1, RBP1, METTL7B, POSTN, CHI3L1, H19, CXCL14, LTF and ENC1) and 9 potential suppressor genes were identified (HR<1; CAMK2A, LINC01088, CDKN2B, LINC00689, TPTEP1, C5orf38, KLRC2, VIPR2, and SMOC1) (**Figure 2B**). **Figure 2C** showed the distribution of error rates in Random Survival Forest model, after which the relative importance of seven genes (H19, CYTOR, IGFBP2, EMP3, KLRC2, C5orf38, and CHI3L1) was established (variable importance>0.25, **Figure 2D**). Multivariate Cox regression analysis was used to develop the cuproptosis-signature, and the CuproptosisScore for each glioma sample was calculated according to the following formula: 0.0621*Expr_H19_+0.0196*Expr_CYTOR_+0.2739*Expr_IGFBP2_+0.0183*Expr_KLRC2_+0.0036*Expr_C5orf38_+0.1406*Expr_CHI3L1_. Heatmap displayed the distribution of six genes in cuproptosis-signature, CuproptosisScore and the clinical characteristics **(** **Figure 2E** **)**. It was clear that a higher CuproptosisScore was linked to higher expressions of H19, CHI3L1, CYTOR, and IGFBP2 and, in contrast, was associated with lower expressions of KLRC2 and C5orf38 **(** **Figure 2E** **)**. In the meantime, IDH mutation status was more likely to be present in glioma patients with lower CuproptosisScore. **(** **Figure 2E** **)**.

### Prognostic potential of cuproptosis-signature

**Figure 3:**
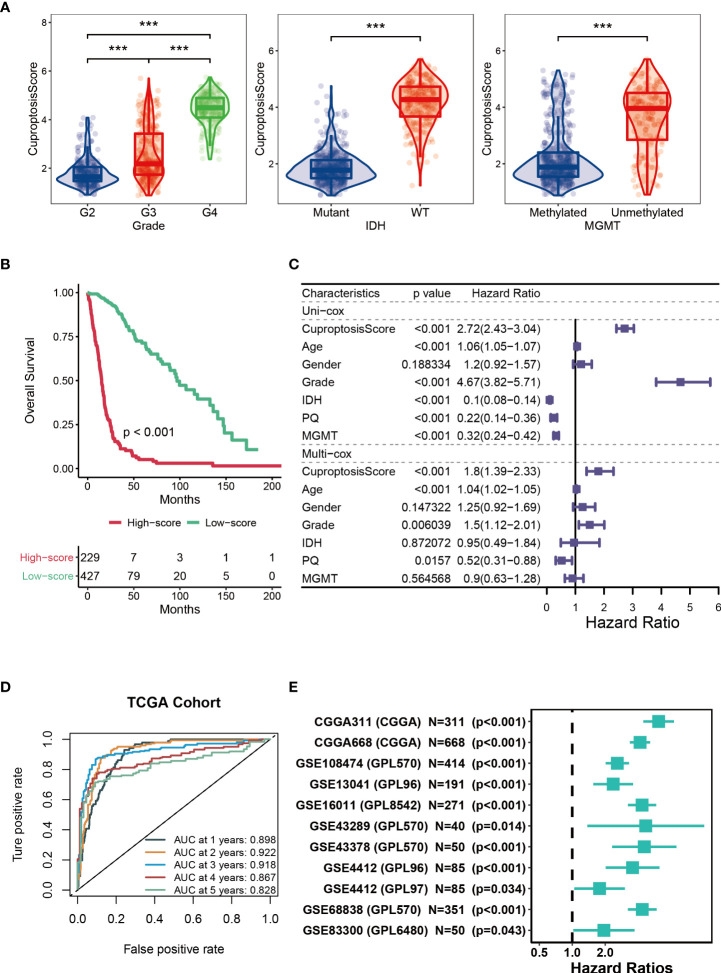
Prognostic potential of cuproptosis-signature. **(A)** The violin figures for comparing the CuproptosisScore of TCGA patients among WHO grades, mutation status and MGMT methylation status. **(B)** Kaplan-Meier survival curve showing survival probability of high-CuproptosisScore or low-CuproptosisScore subgroups. **(C)** The forest figure for Univariate or Multivariate Cox regression analysis of CuproptosisScore and clinicopathologic features. **(D)** The 1-year, 2-year, 3-year, 4-year, and 5-year survival ROC curves are predicted by the cuproptosis-signature. **(E)** Univariate Cox regression analysis of the cuproptosis-signature in 11 external validation data sets. ***P < 0.001.

Next, we analyzed the CuproptosisScore of TCGA patients among WHO grades, mutation status and MGMT methylation status **(** **Figure 3A** **)**. Patients with higher CuproptosisScore had higher WHO grades, while patients with lower grades were more likely to develop IDH mutations or MGMT methylation **(** **Figure 3A** **)**, all of which may explain the significantly better clinical outcomes of patients with lower CuproptosisScore (P<0.001, **Figure 3B** **)**. In addition, Univariate and Multivariate Cox regression analysis of CuproptosisScore and clinicopathologic features demonstrated that both CuproptosisScore and Grade were independent prognostic factors for patients with glioma **(** **Figure 3C** **)**. The survival ROC curves predicted by the cuproptosis-signature showed that the AUCs were all greater than 0.8, indicating the effectiveness of the cuproptosis-signature in predicting prognosis for glioma at the 1-year (AUC=0.898), 2-year (AUC=0.922), 3-year (AUC=0.918), 4-year (AUC=0.867), and 5-year (AUC=0.828) time points **(** **Figure 3D** **)**. Furthermore, we conducted Univariate Cox regression analysis on the OS (overall survival) of glioma patients based on the external validation data sets, and the results showed that HR was greater than 1 in all of the 11 data sets, which further validated the accuracy of our constructed cuproptosis-signature in prognostic prediction **(** **Figure 3E** **)**.

### Genomic mutation analysis for cuproptosis-signature

**Figure 4:**
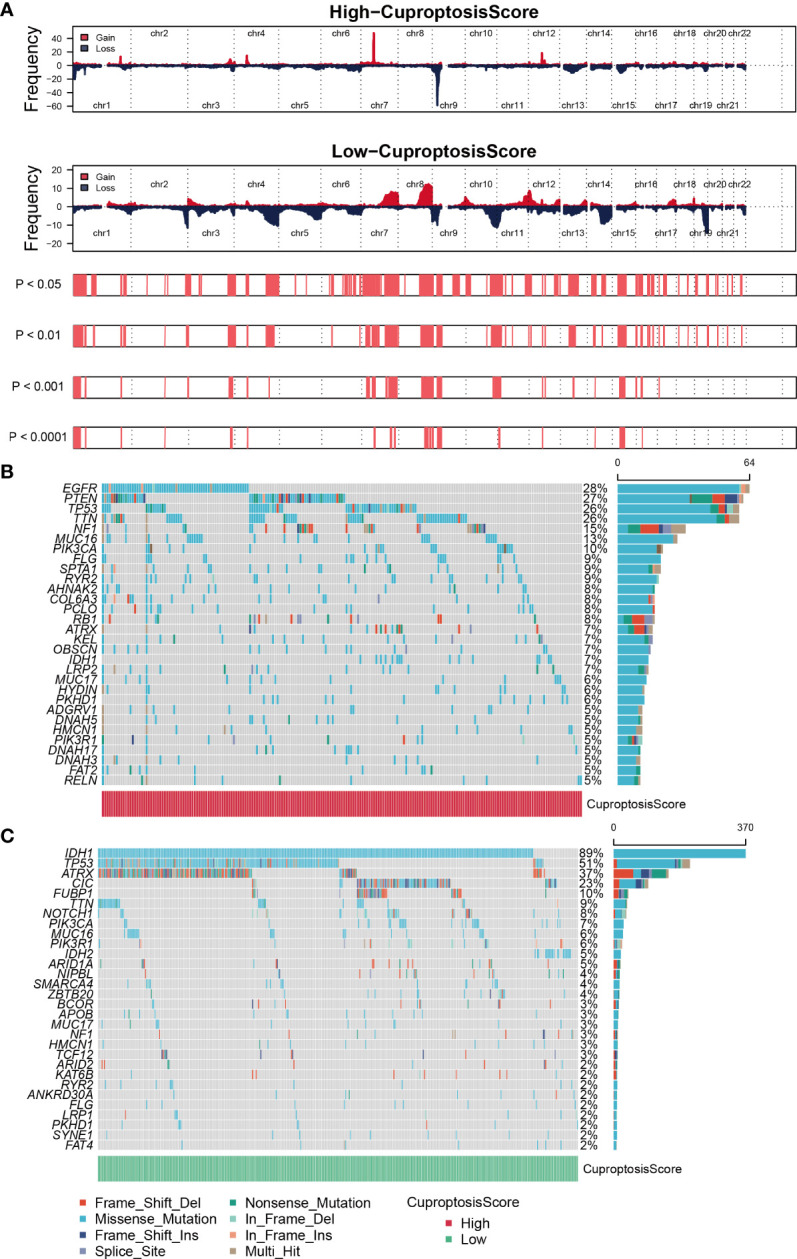
Genomic mutation analysis for cuproptosis-signature. **(A)** Genomic characterization landscape of high-CuproptosisScore or low-CuproptosisScore subgroups. **(B)** Gene mutation frequency in high-CuproptosisScore. **(C)** Gene mutation frequency in low-CuproptosisScore.

GISTIC algorithm was used to assess the genomic characterization landscape between high- CuproptosisScore and low-CuproptosisScore subgroups, which was shown in **Figure 4A**. In patients with high-CuproptosisScore, PIK3CA, MUC16, NF1, TTN, TP53, PTEN, and EGFR had high mutation frequency (over 10%, **Figure 4B**), while in those with low-CuproptosisScore, IDH1, TP53, ATRX, CIC, and FUBP1 had high mutation frequency (over 10%, **Figure 4C**). TP53 had high mutation rates in both groups (26% and 51%, respectively). In agreement with the findings above, the mutation rate of IDH1 was particularly high in the low-CuproptosisScore group, reaching as high as 89% **(** **Figure 4C** **)**.

### Immune status for cuproptosis-signature

**Figure 5:**
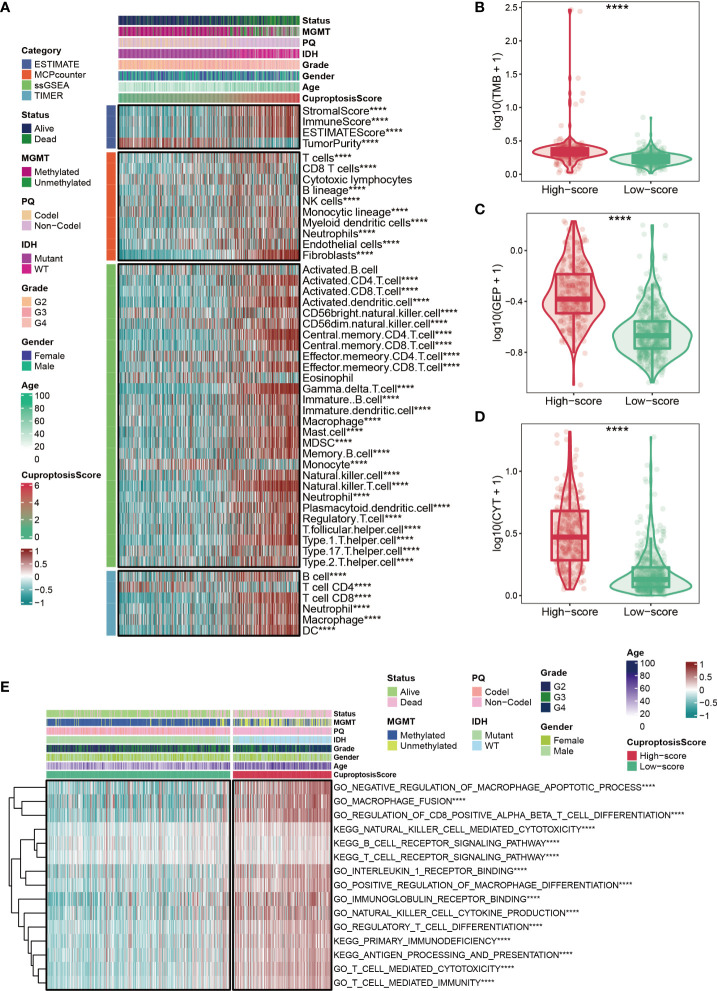
Immune status for cuproptosis-signature. **(A)** The heatmap shows the abundance of infiltrating immune cell populations at different CuproptosisScores. **(B–D)** Glioma patients with high CuproptosisScores had higher levels of TMB **(B)**, GEP **(C)**, and CYT **(D)**. **(E)** The heatmap shows CuproptosisScores, clinical features, and immune-related pathways based on GSVA analysis. ****P < 0.0001.

Based on ESTIMATE, MCPcounter, ssGSEA, and TIMER algorithms mentioned in the Methods section, the heatmap showed the abundance of infiltrating immune cell populations at different CuproptosisScores **(** **Figure 5A** **)**. In general, the level of immune infiltration increased as the CuproptosisScore increased **(** **Figure 5A** **)**. However, it was observed that patients with lower CuproptosisScores had more tumor purity **(** **Figure 5A** **)**. In addition, our results showed that glioma patients with high CuproptosisScores also had higher levels of TMB **(** **Figure 5B** **)**, GEP **(** **Figure 5C** **)**, and CYT **(** **Figure 5D** **)**. GSVA analysis also suggested that patients with high CuproptosisScores were enriched in immune-related pathways, such as negative regulation of macrophage apoptotic process, macrophage fusion, B cell receptor signaling pathway, T cell receptor signaling pathway, and primary immunodeficiency **(** **Figure 5E** **)**.

### Immunotherapy and chemotherapy of cuproptosis-signature

**Figure 6:**
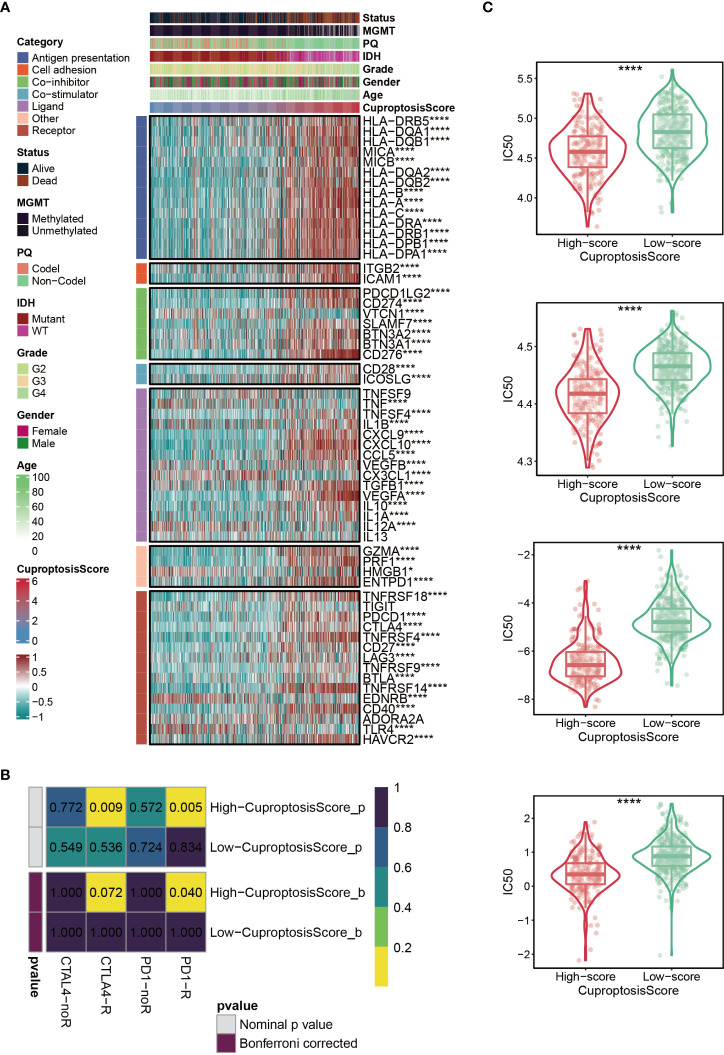
Immunotherapy and chemotherapy of cuproptosis-signature. **(A)** Correlation of CuproptosisScore with seven immunomodulators in gliomas. **(B)** SubMap analysis for cuproptosis-signature in gliomas. **(C)** Box plots of estimated IC50 for several chemotherapeutic agents in the high- or low-CuproptosisScore groups. *P < 0.05; **P < 0.01; ***P < 0.001, ****P < 0.0001.

Immunomodulators (IMs) are closely related to the immunotherapy of malignant tumors, as well, agonists and antagonists for immunomodulators are also being studied (42). The expression of IM-related genes varied across high-CuproptosisScore or low-CuproptosisScore subgroups **(** **Figure 6A** **)**. As the heatmap demonstrated that the level of TNFSF9, IL13, and TIGIT, showed no difference between the two groups, VTCN1, TNF, CX3CL1, IL12A, HMGB1, EDNRB, and TLR4 were highly expressed in the low-CuproptosisScore group, and the remaining genes were highly expressed in the high-CuproptosisScore group **(** **Figure 6A** **)**. In addition, SubMap analysis revealed patients with high-CuproptosisScore may respond to anti-PD-1 therapy **(** **Figure 6B** **)**. This may be due to the high expression of IMs in this group of patients. We also investigated the IC50 values of four chemotherapeutics (Bexarotene, Bicalutamide, Bortezomib, and Cytarabine) between the high- and low-CuproptosisScore groups. Results showed that IC50 values of patients in the high- CuproptosisScore group were lower than those in the low-CuproptosisScore group, suggesting that patients in the high-CuproptosisScore group were more likely to benefit from these four drugs **(** **Figure 6C** **)**.

### The importance of IGFBP2 in TCGA-glioma cohort

**Figure 7:**
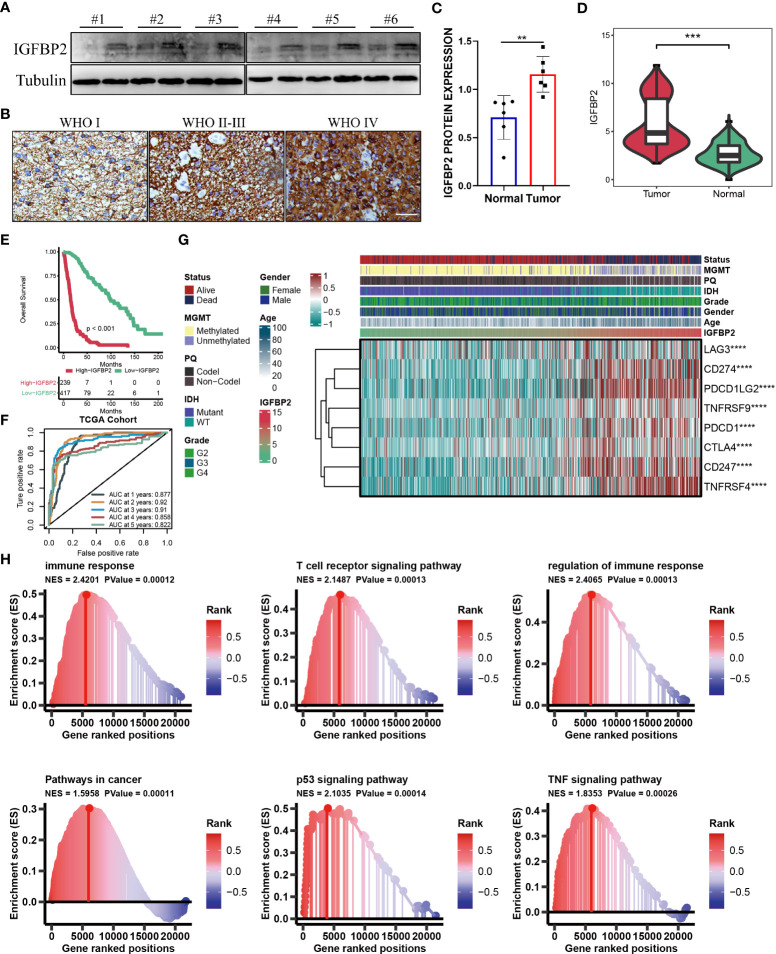
**(A)** WB for IGFBP2 in 3 pairs patients from Nantong cohort. **(B)** Represented IHC for IGFBP2 in three parents with different WHO stage from Nantong cohort. **(C)** Boxplot of IHC for IGFBP2 in six pairs parents from Nantong cohort. **(D)** The expression level of IGFBP2 in glioma sample and the control normal sample. **(E)** Kaplan-Meier survival curve showing survival probability of high- or low-expression IGFBP2. **(F)** The 1-year, 2-year, 3-year, 4-year, and 5-year survival ROC curves are predicted by the expression of IGFBP2. **(G)** The heat map shows the correlation between IGFBP2 and eight immune checkpoints in TCGA. **(H)** GSEA maps of cancer and immune-related signaling pathways positively modulated by IGFBP2. ^∗∗^P < 0.01; ^∗∗∗^P < 0.001; ^∗∗∗∗^P < 0.0001.

The aforementioned results showed that the cuproptosis-signature we created has substantial clinical significance. Next, we randomly selected one gene from this signature, IGFBP2, and explored its important value in gliomas. As is evident from WB and IHC results that IGFBP2 was significantly higher in the six tumor tissues than in the paired adjacent tumor tissues **(** **Figures 7A–C** **)**. The patients with IGFBP2 expression values were listed in the **Supplemental Table S2**. The expression level of IGFBP2 was further compared between the glioma sample and the healthy control sample, it was found that IGFBP2 was significantly overexpressed in the cancer tissue **(** **Figure 7D** **)**. In terms of the survival curve, glioma patients with low expression of IGFBP2 had better survival, indicating that IGFBP2 may be a promoter of the malignant progression of glioma. The 1-year (AUC=0.877), 2-year (AUC=0.92), 3-year (AUC=0.91), 4-year (AUC=0.858), and 5-year (AUC=0.822) survival ROC curves predicted by IGFBP2 revealed that the AUCs were all higher than 0.8, indicating the efficiency of IGFBP2 in predicting prognosis for glioma **(** **Figures 7E, F** **)**. Therefore, IGFBP2 is highly likely to be the oncogenic gene of glioma. The heatmap **(** **Figure 7G** **)** showed that the expression value of IGFBP2 was positively correlated with the expression value of eight immune checkpoints (LAG3, CD274, PDCD1LG2, TNFRSF9, PDCD1, CTLA4, CD247, and TNFRSF4). Moreover, we carried out GSEA analysis to explore cancer and immune-related signaling pathways positively modulated by IGFBP2. We found six signaling pathways **(** **Figure 7H** **)**: immune response, T cell receptor signaling pathway, regulation of immune response, pathways in cancer, p53 signaling pathway, and TF signaling pathway.

## Discussion

Gliomas, especially glioblastoma (GBM), are the most destructive brain tumors within the human nervous system (43). Despite improvements in glioma diagnosis and treatment in recent years, gliomas are still difficult to treat with surgery alone due to their invasive and quickly proliferating nature. Patients with postoperative recurrence have a poor prognosis, with the median survival time only being extended by a few months (44, 45). One of the crucial characteristics of tumor cells is their resistance to cell death. Unrestricted proliferation is typical for tumor cells, and they overcome growth inhibition by resisting death and avoiding being killed by immune cells. However, due to metabolic stress, such as hypoxia and glucose deprivation, necrotizing cell death often occurs in the interior of solid tumors, which affects the occurrence and development of tumors by reshaping the tumor microenvironment. With the discovery of ever-more programmed death modes and the elucidation of associated molecular mechanisms, our understanding of the role of cell death in tumor is constantly updated. Since multiple forms of cell death occur simultaneously in tumors, an in-depth study of cell death on the occurrence and development of tumors can help us better understand their pathogenesis and pave the way for the creation of effective anti-tumor medications. For example, abnormalities of apoptosis pathways play critical roles in tumorigenesis, and tumor cell avoidance of apoptosis has long been thought to lead to primary or acquired therapeutic resistance (46). Necroptosis has both pro-tumor and anti-tumor effects in different types of cancer (13). Inducing necroptosis of tumor cells is an important way to overcome chemotherapy resistance of tumor cells. Finding a novel way to precisely regulate necroptosis might be an essential research target in the field of tumor therapy in the future (47). Numerous pieces of evidence suggest that pyrotopia plays an important role in tumor progression, and inducing pyrotopia has become one of the focuses of cancer immunotherapy (48–51). Ferroptosis is a type of cell death induced by oxidative stress. Cancer cells metabolize more efficiently than normal cells, with a higher ROS load (52) and require large amounts of iron, thus they are more sensitive to ferroptosis than normal cells (53). However, cancer cells also employ additional genetic or epigenetic mechanisms to combat elevated ROS levels, thereby reducing their sensitivity to ferroptosis (54). Therefore, ferroptosis is closely related to the occurrence and development of tumors.

Copper ions can be combined with a variety of proteins or enzymes, as cofactors or structural components, involved in the regulation of energy metabolism, mitochondrial respiration, antioxidant, and other physiological processes (55, 56). The content of copper ions maintains a dynamic balance, which can lead to oxidative stress (55) and abnormal autophagy (56), and thus induce a variety of copper or copper ion-related diseases. Tsvetkov et al. proposed for the first time that a new method of cell death with copper dependence, which was called cuprotosis (14). Several studies had shown that copper metabolism was associated with tumorigenesis, and cancer cells have a higher demand for copper than normal cells (57–60). Wang et al. found that blocking Cu^2+^ transport can cause oxidative stress and decrease cellular ATP levels, which in turn activates AMP-activated protein kinase (AMPK*)*, leading to reduced adipogenesis and inhibiting tumor cell proliferation (61). Studies have confirmed that copper is closely related to the expression level of hypoxia-inducible factor 1α (HIF-1α) (62). The use of copper chelating agent tetrathiomolybdate can significantly reduce the content of Cu^2+^ *in vivo*, and dramatically reduce tumor angiogenesis, restrain tumor growth, and reduce the invasion of breast cancer cells (63). In conclusion, cuprotosis is a novel kind of cellular regulatory death that impacts copper metabolism. The identification of cuprotosis molecular pathways has implications for the mechanism of cuprotosis, cancer drug discovery, and a deeper understanding of copper metabolic diseases. In this study, we included ten cuproptosis-related genes (CRGs): FDX1, LIAS, LIPT1, DLD, DLAT, PDHA1, POHB, MTF1, GLS, and CDKN2A. The correlations among the ten CRGs were primarily positive in TCGA-glioma cohort. In addition, a prognostic signature based on CuproptosisScore was established to explore its prognostic and clinical value in glioma. The current research provides a reference for exploring the mechanism of cuprotosis in the development of glioma.

As for the ten CRGs, the phosphorylation and dephosphorylation of PDHA1 (Pyruvate Dehydrogenase E1 alpha Subunit) are key modulators of deactivation and activation of PDC(Pyruvate Dehydrogenase Complex) (64). It was reported that the increasing level of PDHA1 was observed in the higher grade of glioma and PDHA1 could regulate the migration of glioma cells (65). LINC00665 promoted MTF1 degradation, and MTF1 bound to the promoter region of GTSE1 and transcription promoted GTSE1 expression, which proved that LINC00665/MTF1/GTSE1 axis played an important role in regulating the biological behavior of glioma cells (66). GLS are oncogenic genes of glioma (67, 68) and Qiangzhen Huang et al. found that GLS could regulate the effect of SNAP25 in glioma (66, 69). CDKN2A homozygous deletion was reported to serve as an adverse prognostic factor for IDH-mutant gliomas (70–72). However, to our knowledge, the role of the remaining CRGs in glioma has not been reported in the literature.

The complexity of gliomas is mainly reflected by their molecular heterogeneity. Molecular subtypes can well predict the occurrence and development of glioma polymorphism, which can assist us in developing better treatments (73). Mesenchymal subtypes are particularly malignant compared to other subtypes (neurogenic, canonical, and preneurotic) according to TCGA classification, with recurrent GBM always fatal and often presenting as a mesenchymal phenotype (74–76). In addition, mesenchymal subtypes of gliomas expressed higher levels of angiogenic markers in addition to higher levels of necrosis (74, 77). It has been reported that the transition from the former neural subtype to the mesenchymal subtype is closely associated with treatment resistance and poor prognosis (78). Currently, no fully verified and feasible classification system has been applied to clinical practice, and the glioma classification system needs to be continuously explored and improved. In this study, we determined the optimal number (k=2) of clusters based on R package **(** **Figure 1B** **)**. Furthermore, the reliability of the clustering results was confirmed by PCA analysis **(** **Figure 1C** **)**. Patients with glioma in cluster2 had significantly better clinical outcomes than those in cluster1 **(** **Figure 1D** **)**. Interestingly, it was observed that cluster2 had a higher proportion of IDH mutation status, which may be one of the reasons for the better prognosis of this subcluster **(** **Figure 1E** **)**. In the two subclusters, a total of 27 differentially expressed DEGs were identified **(** **Figure 2A** **)**. When combined with the aforementioned findings, our study sheds light on the need for a new glioma typing system.

Although the WHO classification system has been used for many years to predict the prognosis of patients with glioma, it is occasionally inaccurate due to the heterogeneity of the tumor. In addition to identifying potential biomarkers, new advances in bioinformatics and genome sequencing can help predict cancer patient outcomes and treatment strategies (79, 80). Studies have shown that the prognostic value of a single biomarker is limited, and it is better to integrate multiple biomarkers into a single model (81). For example, three IncRNAs can predict the prognosis of colorectal cancer (CRC) based on a network of metastasis-related competing endogenous RNAs (ceRNA) (82). By extracting TCGA-related data, four IncRNA signals can effectively predict the survival time of lung adenocarcinoma (LUAD) (83). Importantly, recent studies have confirmed the predictive power of some IncRNA prognostic signatures in gliomas, such as immune-associated IncRNAs and autophagy-associated IncRNAs, which have strong prognostic potential for glioma patients (44, 45). In this study, we also used bioinformatics methods to identify a signature containing multiple genes based on CuproptosisScore. Patients with higher CuproptosisScore had higher WHO grades, while patients with lower grades were more likely to develop IDH mutations or MGMT methylation **(** **Figure 3A** **)** and patients with lower CuproptosisScore had the significantly better clinical outcomes **(** **Figure 3B** **)**. In addition, Univariate and Multivariate Cox regression analysis of the signature demonstrated that the cuproptosis-signature was an independent prognostic factor for patients with glioma **(** **Figure 3C** **)**. The survival ROC curves indicated the efficiency of the cuproptosis-signature in predicting prognosis for glioma **(** **Figure 3D** **)**. Furthermore, we conducted Univariate Cox regression analysis on the OS of glioma patients based on the external validation data sets, and the results demonstrated the accuracy of our constructed cuproptosis-signature in prognostic prediction. In conclusion, the signature we have identified has excellent prognostic power **(** **Figure 3E** **)**.

We randomly selected one gene from this signature, IGFBP2, and explored its important value in gliomas. In glioma, IGFBP2 is often involved in the activation of PTEN, AKT and other related pathways, leading to enhanced invasiveness and malignancy (84, 85). Studies have shown that overexpression of IGFBP2 can increase the malignant degree of glioma and up-regulate the expression of invasion protein MMP2, thereby enhancing the invasion ability of glioma cells (86). Previous studies have confirmed that the expression levels of IGFBP2 transcripts and proteins are positively correlated with the malignant degree of glioma, suggesting that IGFBP2 plays an important role in malignant transformation, tumor necrosis and metastasis of glioma (87, 88). In our study, we also found that IGFBP2 is highly likely to be the oncogenic gene of glioma.

However, our study still has several shortcomings. Due to the lack of data of clinical samples collected by us, the prognostic cuproptosis-signature constructed in this study was based on the public database, which may have a certain bias in the source of samples. The hypothesis obtained in this study was not verified by experimental results, and the next step is to be confirmed by various *in vivo* and *in vitro* experiments and larger multicenter studies. In conclusion, this prognostic cuproptosis-signature still needs to be further tested, evaluated and applied in a wide range of clinical settings.

## Data availability statement

The datasets presented in this study can be found in online repositories. The names of the repository/repositories and accession number(s) can be found in the article/**Supplementary Material**.

## Author contributions

WW, ZCL, and MW carried out experiments and analysis. WW, PG, ZCL, MW, ZHL, BW, HH, and CY wrote the manuscript. WW and PG conceived the study. All authors contributed to the article and approved the submitted version.

## Funding

This work was supported by the Medical Scientific Research Project of Jiangsu Provincial Health Commission (H2019058).

## Conflict of interest

The authors declare that the research was conducted in the absence of any commercial or financial relationships that could be construed as a potential conflict of interest.

## Publisher’s note

All claims expressed in this article are solely those of the authors and do not necessarily represent those of their affiliated organizations, or those of the publisher, the editors and the reviewers. Any product that may be evaluated in this article, or claim that may be made by its manufacturer, is not guaranteed or endorsed by the publisher.
